# Effect of different Kinesio tape tensions on experimentally-induced thermal and muscle pain in healthy adults

**DOI:** 10.1371/journal.pone.0259433

**Published:** 2021-11-05

**Authors:** Keith E. Naugle, Jason Hackett, Dania Aqeel, Kelly M. Naugle

**Affiliations:** 1 Department of Kinesiology, School of Health and Human Sciences, Indiana University Purdue University Indianapolis, Indianapolis, Indiana, United States of America; 2 Regenstrief Institute, Indiana University Center for Aging Research, Indianapolis, Indiana, United States of America; 3 Department of Neurology, School of Medicine, Indiana University, Indianapolis, Indiana, United States of America; Leeds Beckett University, UNITED KINGDOM

## Abstract

Athletes and rehabilitation specialists have used Kinesio tape (KT) to help alleviate pain symptoms. Currently, no clear mechanism exists as to why pain is relieved with the use of KT and whether the pain relieving effect is simply a placebo effect. Additionally, the most effective taping parameters (tension of tape) for pain reduction remain unknown. We used quantitative sensory testing to address these key gaps in the KT and pain literature. Using a repeated-measures laboratory design, we examined whether KT applied at different tensions reduces experimentally-induced pain compared to a no tape condition and KT with minimal tension. Heat pain thresholds (HPT’s), pressure pain thresholds (PPT’s), and pressure pain suprathreshold (PPS: 125% of PPT) tests were administered to the forearm prior to and during KT and no tape conditions. Tape was applied to the ventral forearm at 25% of max tension, 75% of max tension, and no tension (placebo). Repeated measures ANOVA’s evaluated the pain outcomes between conditions and across time. KT had no significant effect on PPT’s and HPT’s (p’s >0.05). The ANOVA on PPS revealed that KT applied at 25% of tension significantly reduced pain ratings from the pretest (M = 34.4, SE = 5.5) to post-test 1 (M = 30.3, SE = 4.7) and post-test 2 (M = 30.4, SE = 4.7). No other conditions significantly reduced suprathreshold pressure pain. However, pain ratings at posttest-1 during the no-tape condition (M = 36.4, SE = 5.3) were significantly greater than pain ratings during post-test 1 and post-test 2 of all three tape conditions. In conclusion, the current study revealed that KT applied at low tension is the optimal tension to reduce pressure-evoked muscle pain. Additionally, the results suggested that KT applied at low, high, or no tension may acutely prevent increased muscle sensitivity with repeated pressure stimulation.

## Introduction

In the last decade, the application of Kinesio tape (KT) has emerged as a popular and relatively novel method for reducing acute and chronic musculoskeletal pain and improving muscle and joint function. Kinesio tape is an elastic adhesive tape that mimics the thickness of the skin and can stretch up to 40% of its initial length (i.e., tension of the tape) [1]. Kase et al reports that the tension of the tape lifts the skin to create skin convolutions that aid in blood circulation and lymphatic drainage leading to improved muscle function and a reduction in pain [2]. With the proper application and placement on the body, manufacturers claim Kinesio tape can successfully treat pain in the joints, shoulder, elbow, wrist, back, hips, knee, Achilles tendon, ankle, heel bone, and strained/sore muscles, and headaches [2]. However, the actual evidence supporting Kase et al.’s claims are both limited and mixed.

The existing literature provides mixed results regarding the effectiveness of Kinesio taping for pain reduction [1, 3–6]. One meta-analysis examining KT for the prevention and treatment of sports injuries found that the efficacy of KT in pain relief was trivial [5]. However, another systemic review revealed KT was superior to minimal intervention for musculoskeletal pain relief [3]. Other recent studies have found KT significantly reduces acute non-specific low back pain [7], pain in patients with patellofemoral pain syndrome [8], and delayed onset muscle soreness [1, 3, 9]. In line with the mixed results of KT for pain relief, the mechanisms underlying the pain relieving effects of KT tape are not well understood. Accordingly, several limitations exist in the KT and pain literature. First, the tension (i.e., stretch of the tape when placed on skin) of KT varies considerably between studies, and no study has tested the effectiveness of different tape tensions (i.e., other than tension vs. no tension). Second, many of these studies do not include a sham/placebo condition and a control (no treatment) condition. Third, due to the subjective and variable nature of pain, comparing KT and control conditions in the same participants with objective and quantitative pain assessments would be preferable. However, this type of study design is extremely challenging with clinical pain populations.

One potential promising method for addressing the aforementioned limitations in the KT and pain literature is through the use of quantitative sensory testing (QST). QST is a collection of procedures that assess the perceptual responses to systematically applied and standardized quantifiable sensory stimuli. A large body of literature has used QST to understand the hypoalgesic effects of non-pharmacological interventions in pain-free and chronic pain populations, including exercise [10], virtual reality [11], and massage [12]. QST can be conducted using a variety of stimulus modalities (e.g., thermal, mechanical stimuli) that can vary along important dimensions, including the anatomical site stimulated, the engagement of different nerve fibers and tissues (i.e., cutaneous, muscle tissue), and output measures (threshold vs. pain ratings of suprathreshold stimuli vs. tolerance) [13]. Accordingly, the procedures of the quantitative sensory tests can be manipulated to address a variety of research questions. Also, the acute nature of the pain from QST allows testing of interventions with repeated measures design.

Therefore, we used QST to address key gaps in the Kinesio tape and pain literature. Using a repeated-measures laboratory design, we examined whether KT applied at different tensions reduces experimentally-induced pain compared to two control conditions. Heat pain thresholds, pressure pain thresholds, and suprathreshold pressure pain stimuli (PPS) were administered prior to and during KT and no tape conditions. We purposefully chose these QST tests because they engaged different tissue depths and anatomical structures (i.e., skin, muscle tissue), with the heat pain thresholds engaging cutaneous nociceptors, and the pressure pain tests engaging cutaneous and subcutaneous nociceptors [13]. The PPS test was designed to evoke deep muscle pain [13, 14]. Tape was applied to the ventral forearm at 25% of max tension (low tension), 75% of max tension (high tension), and minimal tension. Overall, we hypothesized that KT applied with tension would exert a greater local pain inhibitory effect compared to KT applied with minimal tension and the no-tape condition, with no differences between the high and low tension KT conditions.

## Methods

### Participants

We recruited and enrolled 16 healthy younger adults (8 males, 8 females) aged 18 years old to 44 years old from within the university setting. A power analysis using G Power 3.0.10 was used to estimate the sample size needed for detecting a within subject difference in pain scores between conditions and tests. With the significance level set at 0.05, power at 0.80, a 0.5 correlation among repeated measures, and an estimated effect size of *f* = .25, the power analyses determined that a total of 13 participants was needed. Exclusion criteria for participants included an open or scabbed wound in the forearm area to be taped, known allergies to the tape or medical adhesive bandages, or if skin irritation develops to the tape. Session exclusion criteria included consuming any caffeinated beverages or OTC pain medications on days of testing prior to testing sessions or active infectious disease or febrile condition (e.g., sinusitis, influenza). If participants reported any of the session exclusion criteria, then the session was rescheduled.

### Procedures

This study was approved by the Indiana University Institutional Review Board (IRB). Participants completed 4 testing sessions held on 4 separate days, that were separated by at least 48 hours. During the first session, the purpose and procedures of the research were explained by the researcher in more detail. Participants read and signed the IRB-approved informed consent form and then completed a questionnaire asking for demographic information. Participants were also asked to complete an 11-point visual analog scale indicating their belief in the ability of Kinesio tape to reduce pain, with 0 indicating the belief that “KT tape will not reduce my pain” and 10 indicating “KT tape will completely remove my pain”. All participants were asked if they have a known allergy to Kinesio Tape or if they have sensitive skin. Those with sensitive skin were administered an allergy test. The allergy test consisted of applying a small patch of Kinesio Tex Tape with “paper-off tension” to the skin of the target tissue. Participants kept this tape on for five minutes. If no skin irritation developed, the tape was removed and the session continued. If skin irritation developed, then the participant was excluded from the study. No participants were withdrawn from this study due to an allergy to the tape.

Once eligibility was established, participants trained in the pain testing procedures to be performed during the experimental testing. Specifically, three trials of pressure pain thresholds and heat pain thresholds were performed on the ventral side of the forearm. Details of these procedures are described below. Session 1 included the above procedures as well as an experimental session. Sessions 2–4 included only the procedures of the experimental session as described below. The order of the experimental sessions was randomized and took place on separate days.

**Fig 1:**
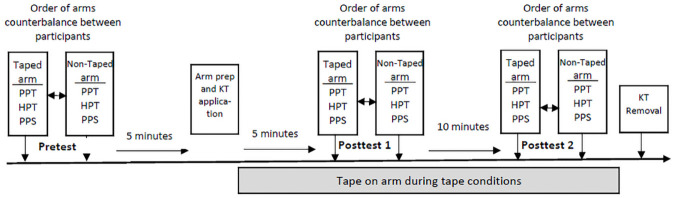
Sequence of events during an experimental session.

Experimental Sessions. For each session, participants were randomized into one of four conditions: Control—no tape, Kinesio Tape applied at 25% tension (low tension), Kinesio Tape applied at 75% tension, (high tension) and Kinesio tape applied with minimal tension. According to Kase et al. [2], the tension of the tape lifts the skin to create skin convolutions or folds in the skin. Therefore, tape with minimal tension should not exert a pain reducing effect and has served as the placebo condition in many KT studies [15–19]. However, it has also been argued that KT applied with minimal tension could also provide stimulation of sensory afferents and therefore, may not serve as a true placebo. As such, we will refer to the KT applied with no tension as the minimal tension condition vs. placebo condition. A researcher (not involved in recruitment, data collection, or administering the interventions) generated counterbalanced session orders and then randomly assigned an order to each participant, with the assignments located in a secure excel sheet. The interventionist had access to the excel sheet to identify the assigned condition each subject was to receive in each session. During each session, the tape was applied for approximately 20 minutes to the ventral side of the right forearm. Quantitative sensory testing as described below was administered 5 minutes prior to the taping (pre-test) and at 5 (post-test 1) and 15–20 (post-test 2) minutes after the tape application. The control session was identical to the sessions with tape application, except no tape was applied. We also included a second control condition during each session, in which QST was administered to the non-taped arm in an identical manner to the taped arm. The control conditions were included to identify any potential effects of repeated pain testing. See Fig 1 for the sequence of events during the experimental sessions.

### Quantitative sensory testing

Pressure pain thresholds (PPT), heat pain thresholds (HPT), and a suprathreshold pressure pain (PPS) test were applied to the skin of each ventral forearm (i.e, between the two tails of the tape on the taped arm and approximately the same location on the non-taped arm) in the aforementioned order. The site for the QST was located between the ulna and radius comprising mainly of muscle and connective tissue. The pressure pain threshold and suprathreshold pain tests were also performed in a slightly different area (within 1 cm) to avoid excessive pain or bruising from repeated pressure pain testing. Each subject was administered two trials of each pain test on each arm (i.e., 2 trials of PPT’s on taped arm and then 2 trials of PPT’s on non-taped arm or vice versa) before moving on to the next pain test, with a 20-sec inter-trial interval. The order of the arms was counterbalanced among participants but remained the same across sessions for individual participants. The researcher administering the QST was blinded to the tension of the kinesio tape. Participants were also not told the tension of the tape.

#### Pressure pain threshold (PPT) testing

A digital, handheld, clinical-grade pressure algometer was used for the mechanical procedures (AlgoMed, Medoc). The experimenter applied a slow constant rate of pressure on the ventral forearm using a .5cm^2^ probe. Participants were instructed to push a button when they first felt the transition from pressure to the sensation of pressure pain. When participants signaled pain, the pressure of the algometer was recorded in kilopascals (kPa) as the PPT. Two trials were administered on each arm during each of the pre and post-tests. Scores were averaged for a single PPT pretest score and a single PPT post-test 1 and post-test 2 score for each arm during each condition.

#### Heat pain threshold (HPT) testing

Heat stimuli was delivered with a 16 x 16 mm thermode (TSA II Neurosensory Analyzer; Medoc, Ltd, Ramat Yishai, Israel) placed and held on the participant’s forearm by the experimenter during testing. The thermode temperature increased from a baseline temperature of 32°C with a rise rate of 0.5 °C/s. Participants were instructed to push a button when they first felt the transition from heat to the sensation of heat pain. When participants signaled pain, the temperature of the thermode was recorded in degrees Celsius as the heat pain threshold (HPT). Two trials were administered on each arm during each of the pre and post-tests. Scores were averaged for a single HPT pretest score and a single HPT post-test 1 and post-test 2 score for each arm during each condition.

#### Pressure pain suprathreshold (PPS) testing

During each separate session, the average PPT pretest value was used to determine a fixed value representing 125% of the average PPT. This pressure amount was used for each subsequent PPS trial during that session. A digital, handheld, clinical grade pressure algometer with a .5cm^2^ probe was used for the PPS testing. The experimenter applied a slow constant rate of pressure on the ventral forearm until a pressure equivalent to 125% of the PPT was reached, at which point the algometer was removed. Participants rated the intensity of pain experienced using a 0–100 numeric rating scale, with “0” identified as “NO PAIN” and “100” representing “INTOLERABLE PAIN”. Two trials were administered on each arm during each of the pre and post-tests. Pain ratings were averaged for a single PPS pretest score and a single PPS post-test 1 and post-test 2 score for each arm during each condition.

### Application of Kinesio tape

The Kinesio tape was applied by a certified athletic trainer on the right forearm. Kinesio Tex Tape, specifically, was used for this experiment (Kinesio Holding Corp, Albuquerque, NM). Prior to applying the tape, the skin in the area where the tape was attached was washed with a moist cloth and then dried with a towel. If needed, hair in the area to be taped was clipped a little shorter with scissors (as recommended by the manufacturers).

**Fig 2:**
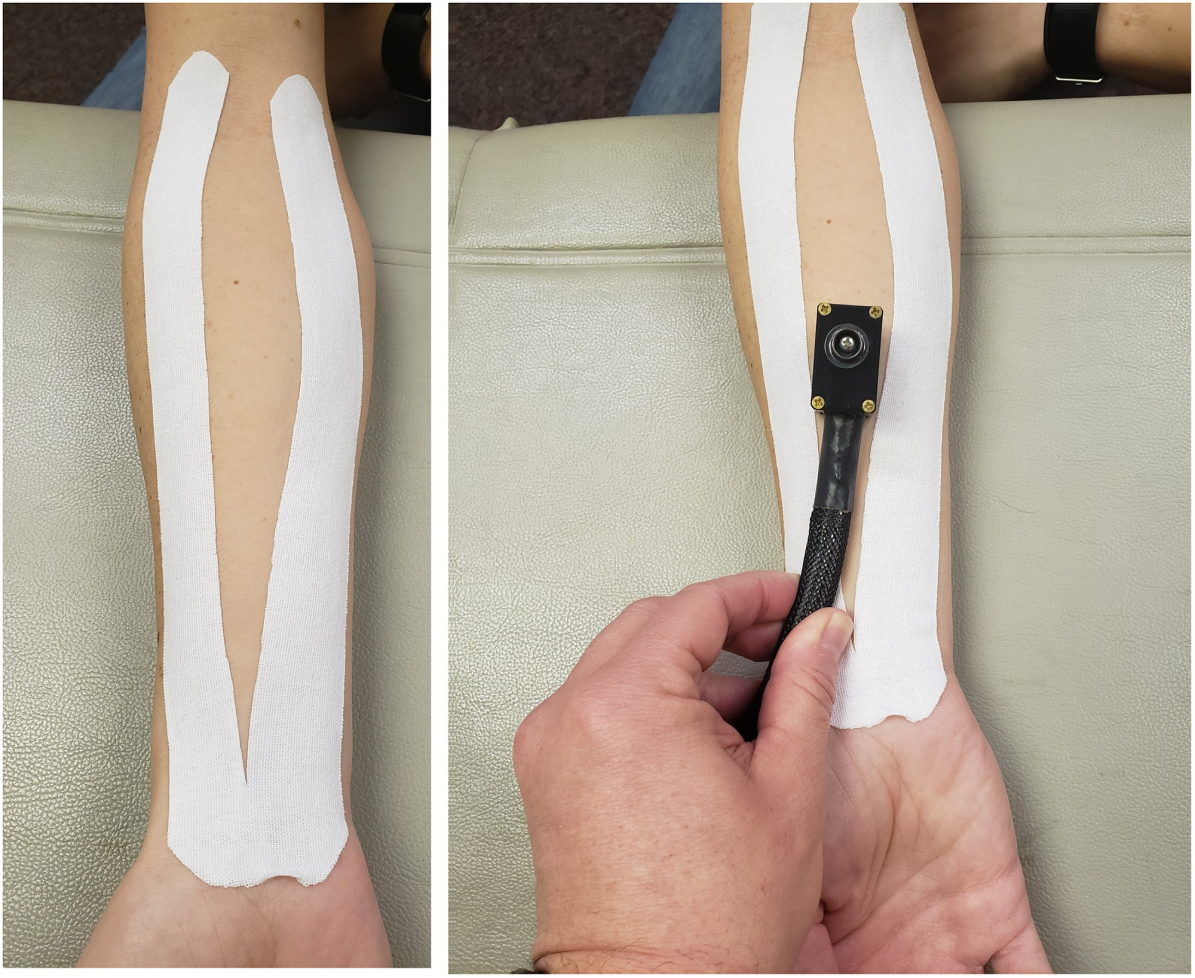
Image showing application of the Kinesio tape on the forearm. The picture on the right shows the placement of the thermode for the HPT test.

Tape was applied as a Y-strip to the ventral forearm as shown in Fig 2 for 20 minutes. The anchor of the KT Y strip was placed near the base of the 5^th^ metacarpal with no tension (i.e., stretch). The tape on the forearm was applied with either minimal tension, 25% of available tension, or 75% of available tension, depending on the condition for that session. The ends were laid down with no tension. Tension refers to the stretch of the tape when applied to the skin. To standardize the amount of tension applied, we used a tape measure to measure the length of the participant’s ventral forearm arm and then calculated the necessary length of the tape (also accounting for the ends that are not stretched) needed to stretch the length of the forearm at the assigned tension. After tape application, the experimenter rubbed the tape to initiate adhesive. The tape was removed after completion of the QST.

### Statistical analyses

All data were analyzed using SPSS v. 26 (SPSS, Inc., Chicago, IL). Descriptive statistics were calculated for all the outcome variables. Shapiro-Wilk’s test of normality indicated that all outcome variables were normally distributed. To assess the reliability of the QST pretests across the four sessions, we computed intraclass correlation coefficients (ICCs) using absolute agreement. ICC coefficients range between 0 and 1.0, with values less than 0.50 indicating poor reliability, values between 0.50 and 0.75 indicating moderate reliability, values between 0.76 to 0.90 indicating good reliability, and above 0.90 indicating excellent reliability [20]. Due to algometer battery issues, one person did not have the PPS test conducted at the last time point during a session; thus, the analyses for the PPS test included only 15 participants. To evaluate the effects of repeated pain testing on the contralateral (un-taped arm) across all sessions, we conducted 4(Condition) x 3(Test) repeated measures ANOVAs on the PPT’s, HPT’s, and PPS tests on the un-taped arm (i.e., left arm). To determine whether the kinesio tape increased pain threshold and decreased pain perception, separate 4(Condition: Tape with minimal tension, Tape at 25%, Tape at 75%, control) x 3(Test: pretest, posttest-1, posttest-2) repeated measures ANOVAs were conducted on the PPT’s, HPT’s, and PPS tests on the taped arm (i.e., right arm). We also wanted to determine the accuracy of the administration of the pressure during the PPS test, which was to be administered at each time point at 125% of the PPT value. Thus, we first calculated the percent deviation of the actual pressure used on the PPS tests relative to the target pressure value (i.e., 125% of PPT). Then, we conducted a 4(Condition: Tape with minimal tension, Tape at 25%, Tape at 75%, control) x 3(Test: pretest, posttest-1, posttest-2) repeated measures ANOVA on the percent deviation values to determine if the values differed by condition and time. Tukey’s post hoc tests were used for all significant main effects or interactions. The p-value for significance was set at p <0.05. The data are presented as Mean ± standard error.

We also report partial eta squared (η_p_^2^), which measures an effect size in ANOVA models. Partial eta squared is measured with the following formula: η_p_^2^ = SS_effect_ / SS_effect_ + SS_error_, with SS_effect_ equaling the sum of squares of an effect for one variable and SS_error_ equaling the sum of squares error in the ANOVA model. The values for η_p_^2^ range from 0 to 1. Higher values indicate a higher proportion of variance that is associated with a given variable in the model after accounting for variance explained by other variables in the model. Partial eta squared values can be interpreted as 0.01 = small effect, 0.06 = medium effect, and 0.14 and higher = large effect size [21].

## Results

### Participants

The average age of participants was 22.3±1.17 (8 males, 8 females). In regards to race, the sample consisted of 12 Caucasians, 2 Hispanics, 1 African American, and 1 Asian. The average belief rating in kinesio tape to reduce pain was 5.4±0.62 (range: 1.2–8.65).

### Reliability of QST tests

The ICC’s indicated that all of the pretest QST tests had good to excellent reliability across sessions: non-taped arm PPT = 0.85, taped arm PPT = 0.83, non-taped arm HPT = 0.94, taped arm HPT = 0.98, non-taped arm PPS = 0.85, taped arm PPS = 0.83.

### Effect of repeated pain testing on non-taped arm

The repeated measures ANOVA conducted on the PPT’s administered to the non-taped arm showed a significant effect of test, p = .026 (η_p_^2^ = .270). Pressure pain threshold significantly increased from the pretest (472.6±51.2) to post-test 2 (518.6±61.0), regardless of condition. The main effect of condition (p = .289, η_p_^2^ = .079) and the interaction (p = .334, η_p_^2^ = .072) were not significant. The repeated measures ANOVA conducted on HPT’s on the non-taped arm demonstrated no significant main effects of condition (p = .909, η_p_^2^ = .013) and test (p = .225, η_p_^2^ = .101) and interaction (p = .312, η_p_^2^ = .079). Similarly, the repeated measures ANOVA conducted on the PPS test administered to the non-taped arm showed no significant effects (condition p = .321, η_p_^2^ = .079; test p = .250, η_p_^2^ = .094; condition x test p = .654, η_p_^2^ = .047). Thus, these results showed only a repeated pain testing effect for PPT’s, in which pain decreased over time within sessions.

### Effect of Kinesio tape on PPT

**Fig 3:**
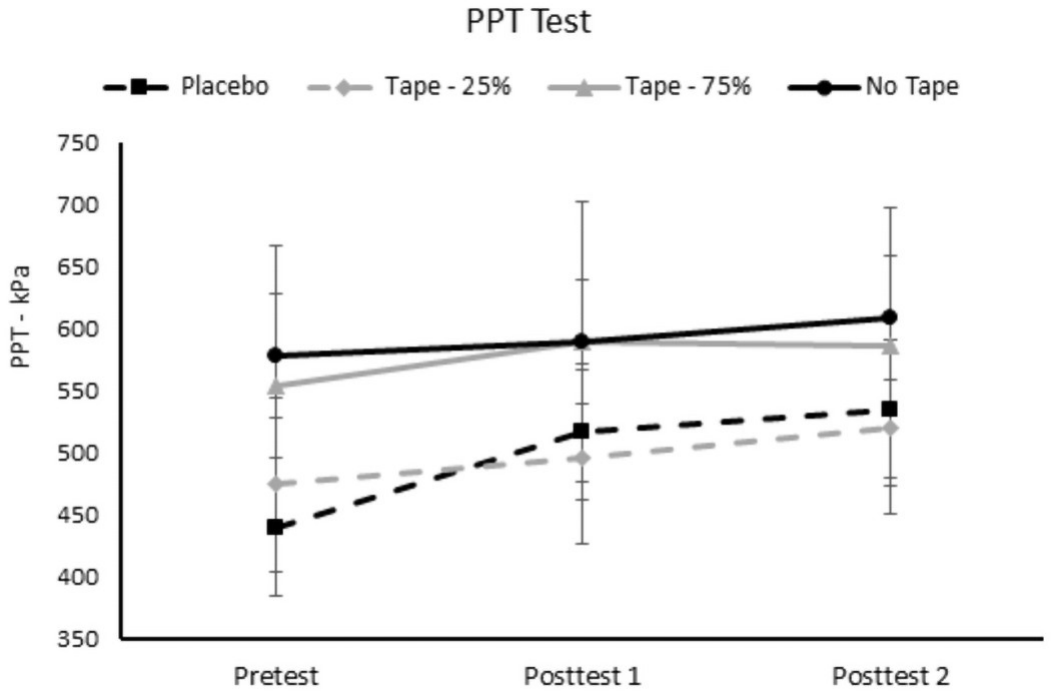
PPT’s on the taped arm for each condition and across evaluation time points. Post-test 1 occurred 5 minutes after tape application. Post-test 2 occurred 15 minutes after tape application. PPT was recorded as the pressure in kilopascals (kPa—y-axis) when the participant first reported pain. PPT = pressure pain threshold.

The repeated measures ANOVA conducted on the PPT’s administered on the taped arm revealed a significant effect of test, p = .027 (η_p_^2^ = .258). The follow-up tests indicated that pressure pain threshold significantly increased from the pretest (511.62±67.48) to post-test 2 (562.31±73.65), regardless of condition. The main effect of condition (p = .341, η_p_^2^ = .068) and condition x time interaction were not significant (p = .143; η_p_^2^ = .099). See Fig 3 for the PPT’s on the taped arm across time and conditions.

### Effect of Kinesio tape on HPT

**Fig 4:**
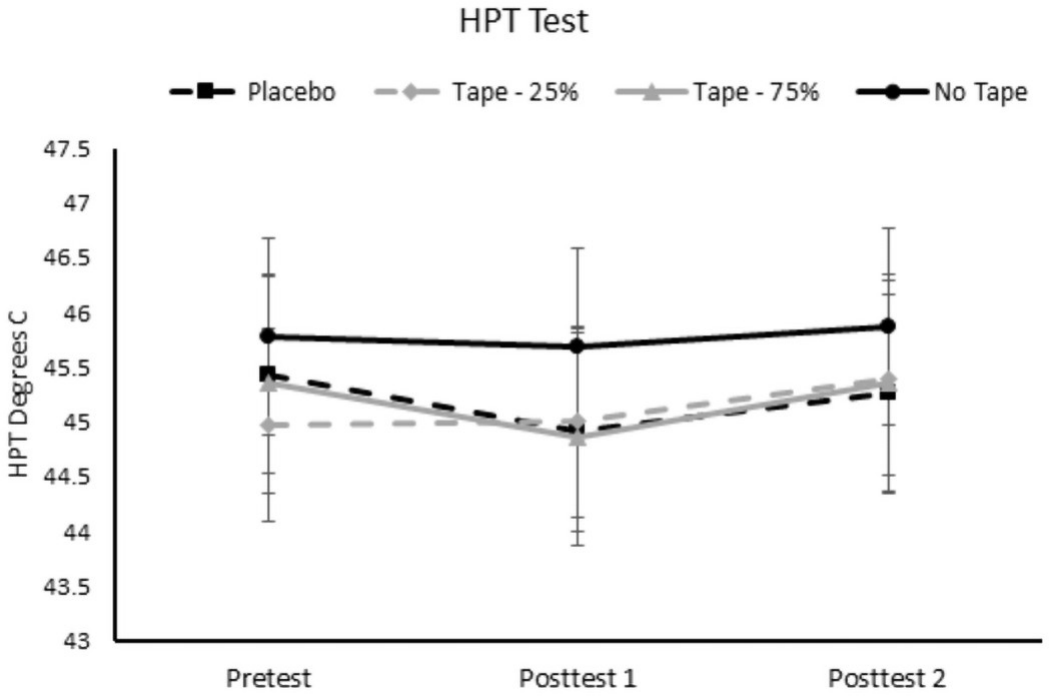
HPT’s on the taped arm for each condition and across evaluation time points. Post-test 1 occurred 5 minutes after tape application. Post-test 2 occurred 15 minutes after tape application. HPT was recorded as the temperature of the thermode in degrees Celsius (y-axis). HPT = heat pain threshold.

The repeated measures ANOVA conducted on the HPT’s administered on the taped arm demonstrated no significant main effects of condition (p = 0.175, η_p_^2^ = .110) and test (p = 0.281; η_p_^2^ = .081) or interaction (p = 0.581; η_p_^2^ = .05). See Fig 4 for the HPT’s on the taped arm across time and conditions.

### Effect of Kinesio tape on PPS

**Fig 5:**
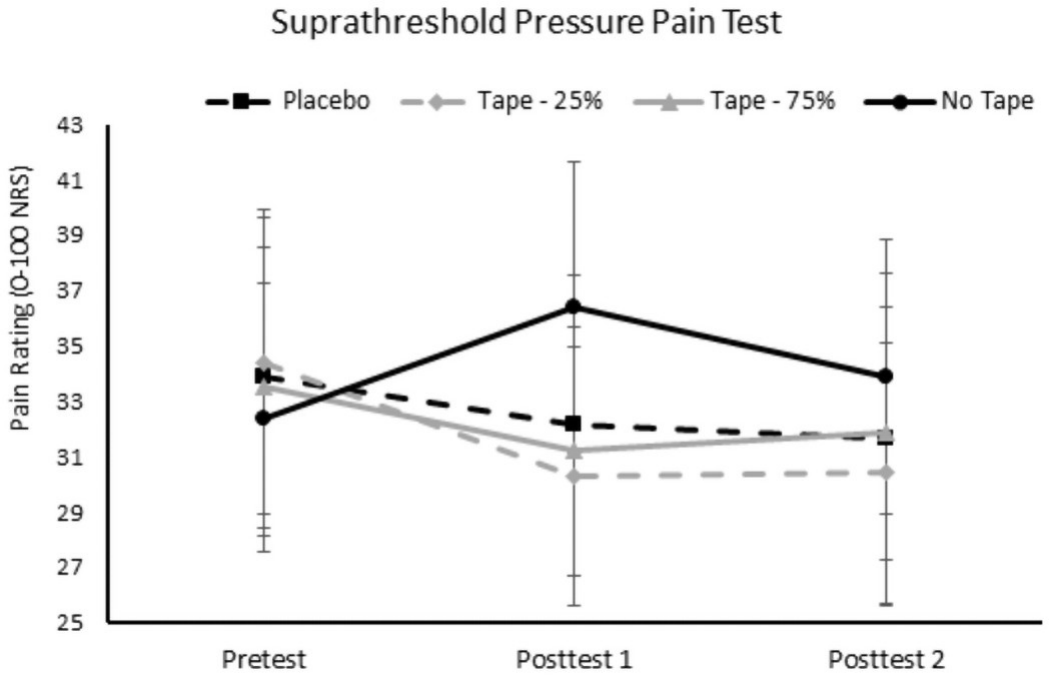
PPS’s on the taped arm for each condition and across evaluation time points. Post-test 1 occurred 5 minutes after tape application. Post-test 2 occurred 15 minutes after tape application. The outcome measure for the PPS test was the intensity of pain ratings on a 0–100 numeric rating scale (y-axis). PPS = pressure pain suprathreshold.

The analysis showed non-significant main effects of condition (p = .591, η_p_^2^ = .047) and test (p = .450, η_p_^2^ = .042). However, the condition x test interaction was significant, p = .018 (η_p_^2^ = .164). The follow-up tests revealed that KT applied at 25% of tension significantly reduced pain ratings from the pretest to post-test 1 and post-test 2. No other conditions significantly reduced pain. However, in the no-tape condition, pain ratings significantly increased from the pretest to post-test 1. Additionally, pain ratings at post-test 1 during the no-tape condition were significantly greater than pain ratings during post-test 1 and post-test 2 of all three tape conditions. See Fig 5 for the pain ratings of the PPS tests on the taped arm across time and conditions.

We also examined the accuracy of the administration of the pressure during the PPS test. For the taped arm, the analysis conducted on the percent deviation from the target pressure on the PPS tests indicated no significant main effects of condition (p = 0.67) or test (p = 0.96) or condition x test interaction (p = 0.49). The data indicated that on average the percent deviation from the target pressure on the taped arm was 1.42% [S.E. = 0.53, 95% Confidence Intervals (CI’s): 0.28, 2.55]. For the non-taped arm, the repeated measures ANOVA revealed a significant main effect of test (p = 0.042), with the pretest percent deviation (2.3±0.7) significantly higher than the post-test 2 percent deviation (1.43±0.5). The main effect of condition (p = 0.64) and condition x test interaction (p = 0.69) were not significant. The average percent deviation from the target pressure on the non-taped arm was 1.82%±0.57 (95% CI’s: 0.59, 3.04).

## Discussion

The current study used QST in healthy younger adults to enhance the understanding of Kinesio tape’s pain relieving effects and mechanisms of action. The use of acute experimental pain assessment and repeated measures design allowed assessment of KT applied at different tensions compared to control conditions in the same individuals. The primary finding of this study was that KT applied at 25% of max tension reduced pressure-evoked muscle pain, whereas KT applied with minimal tension and 75% tension produced no pain relieving effects.

The present study included quantitative sensory tests that varied in stimulus modality (e.g., thermal, mechanical stimuli) and output measures (thresholds vs. pain ratings of suprathreshold stimuli). Thus, the different QST tests engaged different tissue depths and anatomical structures (i.e., skin, muscle tissue), with the HPT’s engaging cutaneous nociceptors, and the pressure pain tests engaging cutaneous and subcutaneous nociceptors [13]. The PPS test was designed to evoke deep muscle pain [13, 14]. Our results revealed that KT at 25% tension significantly reduced muscle pain evoked by the PPS test. However, KT did not alter HPT’s or PPT’s. These results indicate that KT’s pain-relieving mechanism primarily worked via reducing pain perception on the deep muscle tissue. In a similar study, Liu et al examined the acute effect of different kinesio taping conditions (i.e., Y-strips vs. fan-strips) on PPT’s and pain during electrical stimulation in healthy women [22]. The tape in both KT conditions was applied at 10–15% tension on the lower back. The results indicated that only the fan-strip KT elicited higher PPT’s compared to the placebo tape, and no differences existed between conditions for the electrical stimulation pain. Thus, similar to the current study, Liu et al found no acute effects of a KT Y-strip on PPT’s. Another study evaluated the effects of KT applied to the lumbar region on a battery of QST tests in healthy individuals [23]. KT applied at 20% tension and a standard “rigid” tape (non-KT tape) significantly increased mechanical detection and pain thresholds compared to a sham condition. However, HPT’s increased across all three conditions and no changes were found for PPT’s. Because the study did not have a control condition, the authors could only speculate on whether the increase in HPT’s was due to habituation or placebo effect. Importantly, these collective studies suggest that the type of taping method and noxious stimulation are likely important factors to consider in the pain relieving effects of KT.

The optimal tape tension of KT for pain relief is not clear. One recent meta-analysis of 17 studies on KT for musculoskeletal pain reported that the tension of KT applied to the skin ranged from 12.5% to 70% stretch across these studies [3]. While several studies in this review compared KT applied with tension to no tension (placebo), no studies compared the effectiveness of different tensions (other than placebo). Accordingly, clinical studies of KT are limited in their ability to assess the effectiveness of multiple tape tensions, especially in the same individual. To the best of our knowledge, the current study is the first to compare the analgesic effects of three different KT tensions with a repeated measures design. The results indicated that only KT applied at 25% tension exerted a significant acute pain relieving effect on pressure evoked muscle pain. This result is in accordance with a meta-analysis on KT and musculoskeletal pain that found lower effect sizes for pain reduction in studies that applied higher tension [3]. Additionally, Kase et al. suggested that KT applied with too much tension may attenuate the beneficial effects of KT or even lead to a pain increase, although no evidence was actually provided to support this assertion [2]. While we did not find increased pain with the high tension KT, our results did provide experimental evidence that low tension KT is optimal for pressure pain reduction. Future experimental research is needed to clarify the effect of KT applied at different tensions on clinical musculoskeletal pain conditions.

Several mechanisms have been proposed to explain KT’s pain-relieving effect. One potential mechanism includes lifting the skin to create space between the superficial skin and its associated underlying connective tissues [24, 25]. Lifting of the skin could improve blood circulation and lymph flow to remove pain substances and decrease pressure on subcutaneous nociceptors [26]. However, this mechanism cannot explain why KT applied at 25% versus 75% decreased pressure evoked muscle pain. Others have proposed KT works via the gate control theory [22, 23, 27]. According to the gate control theory, stimulation of non-nociceptive fibers (Aβ) inhibits the transmission of nociceptive input from small diameter nerve fibers (Aδ, C) to the spinal cord, thereby reducing pain [28, 29]. Thus, non-painful mechanical stimulation of the skin via KT could inhibit or interfere with signals from pain fibers, thereby decreasing pain. Perhaps the gate control theory could explain why the low tension condition resulted in a pain-relieving effect compared to the no tension and high tension KT conditions. For example, KT applied with no tension may not have provided enough sensory stimulation to activate the “gate”, while KT applied with high tension may have irritated the skin and activated nociceptive nerves. On the other hand, KT applied with low tension may have provided sufficient non-nociceptive mechanical stimulation to reduce ongoing nociceptive transmission to the spinal cord. However, these hypotheses regarding the mechanisms are purely speculative and should be followed up with future research.

Importantly, our results also demonstrated that pressure pain ratings increased in the no tape condition from the pretest to posttest-1, potentially indicating increased pressure sensitivity with repeated stimulation. Interestingly, while KT at 75% and minimal tension did not significantly reduce pressure pain in the current study, pain ratings at posttest 1 were significantly lower in all taping conditions compared to the no tape condition (no difference in pretest between all conditions). Thus, it is possible that the KT conditions, regardless of tension, prevented the deep tissue from becoming more sensitive to pressure pain. This finding is similar to Kalichman and colleagues who investigated the immediate and short-term effects of KT on myofascial trigger point sensitivity in healthy adults using PPT’s [30]. Kalichman et al found that in the control condition PPT’s lowered over time (increased sensitivity) on the trigger points. However, KT at 30% tension placed above the myofascial trigger points prevented this increase in pressure sensitivity immediately after the application. The Kalichman study results are likely more similar to our PPS data than PPT data because in the Kalichman study PPT’s were administered on trigger points (i.e., hyperirritable spots), whereas the pain tests in the current study were not administered on sensitive trigger points.

The evidence on whether KT reduces pain through a placebo effect is mixed [1, 3, 6]. In most studies, placebo KT is administered as the KT with no tension, as was done in the current study. Studies of clinical [16, 31] and experimentally induced pain [19, 22] have found that KT applied with tension significantly reduces pain compared to placebo KT conditions (i.e., KT with no tension, tension-free KT applied only at the ends, non-KT tape). However, Parreira et al recently conducted a large randomized control trial designed to test whether KT applied with 10–15% tension was more effective in reducing pain than a placebo KT application (KT with no tension) in individuals with chronic low back pain [18]. Interestingly, both groups experienced significant pain reduction, challenging the theory that the creation of skin convulsions are needed for pain relief with KT. This result is in accordance with a recent meta-analysis finding that KT is not superior to sham KT in pain relief for individuals with musculoskeletal conditions [32]. In the current study, the KT with minimal tension condition did not significantly reduce pain sensitivity or perception during any of the QST tests, suggesting that KT’s pain relieving action maybe more than a placebo effect. However, as mentioned previously, all the taping conditions in the present study prevented an increase in pressure pain sensitivity during the PPS test. Perhaps, the sensory stimulation provided by the tape regardless of tension is sufficient to prevent increased muscle sensitivity with repeated pressure stimulation. Future research is still needed to determine if patient expectations contribute to KT’s pain relieving effect. In the current study, participants’ belief ratings in KT to reduce pain ranged from 1.2 to 8.65 on a 0–10 scale. Future studies could compare the effects of KT on pain between individuals that have a high belief in KT to reduce pain and those who have a low belief.

Several limitations existed in this study. First, participants and the experimenters were blinded to the tension of the tape but could not be blinded to the no-tape condition. Additionally, while the participants were never told that tape would be applied with different tensions, it is possible that participants could feel that tape was administered at different tensions during each session. However, participants were likely naïve to any expectations regarding the effects of KT applied at different tensions. Second, this study only included healthy subjects without clinical pain; therefore, the results may not apply to other pain conditions. However, most individuals with musculoskeletal pain report pain from deep tissues, including muscle and other soft tissues. Thus, the PPS test of the muscles is likely clinically relevant. Third, this study only observed the short-term effects of KT on acute pain. Effects of longer tape applications on chronic pain could yield different results. Lastly, we had to assess pain sensitivity and perception on the forearm in between the tales of the KT vs on the taped skin because the QST tests needed to be administered directly on the skin. However, studies have found that KT causes measurable tissue deformations and changes in local skin temperature in the adjacent skin/tissues to the site of tape application [26, 33]. Additionally, other studies have found pain relieving effects of KT on experimental pain on the adjacent skin areas to site of tape application [23, 34]. Nonetheless, it is possible that KT could have stronger pain relieving effects directly on the taped vs. non-taped skin.

In conclusion, the current study suggests that KT applied at low tension provides a greater pain relieving effect compared to no tape and KT applied at no tension and high tension for pressure-evoked muscle pain. Additionally, the results suggested that KT applied at low, high, or no tension may acutely prevent increased muscle sensitivity with repeated pressure stimulation. Importantly, our data suggest that KT’s pain relieving effects are likely more than a placebo effect. Future studies are needed to determine the mechanisms through which KT at low tension reduces muscle pain, as well as the mechanisms through which KT at any tension prevents increased pressure sensitivity with repeated muscle stimulation.

## Supporting information

S1 File Dataset. (PDF)

S2 File Visual analog scale to measure belief in the ability of KT to reduce pain. (PDF)
