# Supplementary material for: Effect of different Kinesio tape tensions on experimentally-induced thermal and muscle pain in healthy adults
Source: PLoS One. 2021 Nov 5;16(11):e0259433. doi: 10.1371/journal.pone.0259433 (PMC8570489; doi:10.1371/journal.pone.0259433)
Supplement: S1 File — (PDF) [file pone.0259433.s001.pdf]

AGE	SEX	RACE	BELIEF_RATING	KT-NO TENSION_PrePPT_LEFT ARM	KT-NO TENSION_PrePPT_RIGHT ARM	KT-NO TENSION_PreHPT_LEFT ARM	KT-NO TENSION_PreHPT_RIGHT ARM	KT-NO TENSION_PPS_TARGET PRESSURE_LEFT ARM
23.00	1.00	3.00	0.30	587.40	793.40	46.10	47.10	733.50
20.00	2.00	3.00	7.50	693.35	242.87	43.55	43.70	865.90
21.00	1.00	3.00	2.20	365.80	370.70	41.10	44.60	457.20
21.00	1.00	3.00	7.00	374.60	346.20	47.30	47.80	468.30
21.00	2.00	3.00	8.65	375.35	389.10	44.95	45.35	468.00
22.00	1.00	3.00	7.35	701.70	476.30	48.95	49.80	877.00
20.00	1.00	4.00	6.70	349.80	370.45	44.10	40.65	435.50
27.00	1.00	3.00	4.80	244.05	229.30	43.00	44.75	305.00
19.00	2.00	3.00	3.80	502.25	627.10	46.65	46.40	627.10
30.00	2.00	3.00	8.60	509.85	525.30	41.60	43.15	637.20
34.00	1.00	1.00	4.30	549.65	760.70	47.00	49.60	687.30
21.00	1.00	3.00	1.20	560.85	462.95	48.05	48.40	701.10
18.00	2.00	2.00	6.00	96.90	99.45	38.45	37.75	121.30
20.00	2.00	3.00	6.10	168.30	214.10	43.30	41.85	210.10
22.00	2.00	3.00	1.70	499.65	560.85	46.50	48.40	624.60
18.00	2.00	4.00	5.60	564.95	575.10	46.40	47.60	706.70

KT-NO TENSION_PPS_TARGET PRESSURE_RIGHT ARM	KT-NO TENSION_PrePPS_LEFT ARM	KT-NO TENSION_PrePPS_RIGHT ARM	KT-NO TENSION_PrePPS_ACTUAL PRESSURE_LEFT ARM	KT-NO TENSION_PrePPS_ACTUAL PRESSURE_RIGHT ARM	KT-NO TENSION_POST1_PPT_LEFT ARM	KT-NO TENSION_POST1_PPT_RIGHT ARM	KT-NO TENSION_POST1_HPT_LEFT ARM	KT-NO TENSION_POST1_HPT_RIGHT ARM
759.00	5.00	5.00	758.05	763.90	728.60	671.75	45.40	47.00
621.70	23.00	16.00	871.85	623.70	642.35	501.15	43.20	44.30
463.40	25.00	20.00	467.30	467.75	321.65	449.15	43.05	42.80
432.70	7.00	5.00	478.60	447.20	392.25	449.15	49.05	48.65
486.00	72.50	77.50	474.35	506.65	460.60	425.35	44.70	44.80
595.00	30.50	21.50	931.95	614.45	646.80	576.25	49.25	48.50
462.80	15.00	12.50	448.80	470.35	344.90	355.50	42.25	39.35
286.60	40.00	38.00	329.30	278.35	241.05	275.40	43.70	45.15
783.10	45.00	50.00	644.95	789.80	560.85	639.85	46.25	46.30
655.70	43.50	28.00	637.30	657.70	522.65	718.90	41.85	44.50
951.40	62.50	57.50	674.00	953.40	657.70	794.35	48.65	49.60
578.70	5.50	6.00	691.35	615.90	611.80	599.55	46.55	47.80
123.40	61.50	60.00	121.35	122.90	107.05	96.35	38.90	38.10
267.70	45.00	37.50	214.10	265.10	183.55	183.55	39.50	36.05
701.10	43.50	44.00	649.55	690.30	473.10	637.35	48.65	48.10
718.90	41.00	46.00	709.70	725.05	778.05	897.35	46.05	47.55

KT-NO TENSION_POST1_PPS_LEFT ARM	KT-NO TENSION_POST1_PPS_ACTUAL PRESSURE_LEFT ARM	KT-NO TENSION_POST1_PPS_RIGHT ARM	KT-NO TENSION_POST1_PPS_ACTUAL PRESSURE_RIGHT ARM	KT-NO TENSION_POST2_PPT_LEFT ARM	KT-NO TENSION_POST2_PPT_RIGHT ARM	KT-NO TENSION_POST2_HPT_LEFT ARM	KT-NO TENSION_POST2_HPT_RIGHT ARM	KT-NO TENSION_POST2_PPS_LEFT ARM
5.00	709.00	5.00	760.05	752.15	845.35	44.80	46.55	5.00
29.50	867.85	22.50	632.50	732.55	560.00	44.10	44.25	31.00
22.50	465.80	15.00	476.60	372.65	383.45	44.30	45.75	15.00
4.50	480.50	2.50	467.75	462.85	569.75	48.20	47.95	6.50
52.50	479.20	65.00	487.05	434.15	507.65	45.30	45.30	65.00
36.50	906.25	24.00	607.60	597.80	449.80	49.60	49.35	31.50
15.00	#NULL!	17.50	#NULL!	412.70	380.00	43.35	40.10	10.00
40.00	305.75	34.00	294.00	255.80	227.35	44.55	45.05	38.00
65.00	665.35	40.00	810.70	558.30	706.15	47.25	46.25	50.00
29.50	644.95	31.00	652.60	512.45	650.05	41.15	42.85	31.00
70.00	689.30	71.50	948.30	572.25	826.00	46.80	49.35	72.50
8.50	721.95	6.50	577.15	597.60	548.65	49.30	47.65	10.50
60.00	117.80	52.50	122.90	102.00	99.95	38.45	36.85	57.50
50.00	209.00	45.00	270.20	203.90	173.35	41.90	39.60	42.50
38.50	641.40	40.50	704.65	595.55	713.80	49.70	49.15	28.50
39.00	703.60	32.00	720.95	947.30	917.75	47.70	48.35	30.00

KT-NO TENSION_POST2_PPS_ACTUAL PRESSURE_LEFT ARM	KT-NO TENSION_POST2_PPS_RIGHT ARM	KT-NO TENSION_POST2_PPS_ACTUAL PRESSURE_RIGHT ARM	KT-25% TENSION_PrePPT_LEFT ARM	KT-25% TENSION_PrePPT_RIGHT ARM	KT-25% TENSION_PreHPT_LEFT ARM	KT-25% TENSION_PreHPT_RIGHT ARM	KT-25% TENSION_PPS_TARGET PRESSURE_LEFT ARM	KT-25% TENSION_PPS_TARGET PRESSURE_RIGHT ARM
727.70	5.00	747.25	637.40	834.55	46.60	46.30	797.30	1042.40
866.90	26.00	632.50	502.10	479.55	39.80	42.60	627.60	599.20
463.85	5.00	477.55	411.85	339.25	45.35	46.10	514.80	433.00
479.55	3.00	438.35	286.35	288.35	47.15	45.45	357.90	360.40
475.30	65.00	496.85	447.85	448.85	44.25	45.35	560.00	561.00
898.40	22.00	586.05	517.40	558.60	49.40	49.30	647.00	698.00
#NULL!	18.50	#NULL!	663.91	665.87	42.20	40.05	829.64	831.60
327.35	39.50	295.00	205.80	202.85	43.70	43.35	257.25	253.60
642.40	45.00	803.05	260.05	268.20	46.70	45.75	325.30	334.50
639.85	33.00	660.25	386.45	403.80	44.60	45.85	483.30	504.80
687.30	76.00	938.15	463.95	741.30	47.25	48.25	580.20	925.90
726.00	5.50	602.65	370.15	421.05	46.05	47.00	461.90	526.20
121.85	52.50	121.85	141.70	122.40	40.50	40.40	141.70	122.40
209.00	55.00	270.20	132.60	158.10	36.45	36.85	165.70	196.80
642.40	27.00	710.75	561.85	721.95	48.60	48.60	702.30	902.40
704.65	22.50	724.00	801.50	935.10	48.85	48.45	1002.40	1168.60

KT-25% TENSION_PrePPS_LEFT ARM	KT-25% TENSION_PrePPS_RIGHT ARM	KT-25% TENSION_PrePPS_ACTUAL PRESSURE_LEFT ARM	KT-25% TENSION_PrePPS_ACTUAL PRESSURE_RIGHT ARM	KT-25% TENSION_POST1_PPT_LEFT ARM	KT-25% TENSION_POST1_PPT_RIGHT ARM	KT-25% TENSION_POST1_HPT_LEFT ARM	KT-25% TENSION_POST1_HPT_RIGHT ARM	KT-25% TENSION_POST1_PPS_LEFT ARM
5.00	7.50	780.60	1037.55	720.80	768.80	45.20	46.70	5.00
35.00	42.50	632.55	602.15	456.00	426.60	42.25	43.65	24.50
30.00	15.00	528.60	433.50	318.70	388.30	43.00	46.40	25.00
8.00	3.00	386.35	386.40	308.90	298.15	48.05	47.00	5.50
69.00	73.50	560.60	579.20	456.70	391.55	45.00	44.35	75.00
18.50	26.50	721.25	707.55	539.00	496.85	49.40	49.75	34.50
12.50	15.00	834.55	842.39	812.00	764.92	43.70	43.00	15.00
46.50	41.50	269.50	261.65	320.45	226.40	44.10	41.95	34.00
47.50	45.00	335.45	337.50	271.25	268.20	46.50	45.45	50.00
49.50	41.00	484.35	512.90	293.70	384.40	44.25	44.55	40.50
55.00	60.00	571.05	909.55	526.15	680.20	47.65	48.50	55.00
16.50	12.50	478.25	559.80	433.40	467.00	45.70	46.10	14.00
65.00	62.50	135.15	121.85	117.75	117.75	40.95	40.00	57.50
52.50	37.50	183.50	203.90	153.00	193.70	39.20	36.45	27.50
22.00	29.00	725.00	915.70	679.15	870.80	49.15	48.60	30.50
44.50	46.00	1004.45	1164.50	976.90	1200.20	47.55	47.65	27.50

KT-25% TENSION_POST1_PPS_ACTUAL PRESSURE_LEFT ARM	KT-25% TENSION_POST1_PPS_RIGHT ARM	KT-25% TENSION_POST1_PPS_ACTUAL PRESSURE_RIGHT ARM	KT-25% TENSION_POST2_PPT_LEFT ARM	KT-25% TENSION_POST2_PPT_RIGHT ARM	KT-25% TENSION_POST2_HPT_LEFT ARM	KT-25% TENSION_POST2_HPT_RIGHT ARM	KT-25% TENSION_POST2_PPS_LEFT ARM	KT-25% TENSION_POST2_PPS_ACTUAL PRESSURE_LEFT ARM
807.10	10.00	1015.00	708.05	779.65	44.25	45.15	5.00	813.00
636.45	36.50	605.05	543.30	527.55	43.60	45.30	32.00	634.50
528.60	15.00	430.50	380.50	386.35	45.50	48.20	20.00	522.70
386.40	2.00	371.65	357.95	401.05	48.20	48.10	4.00	368.75
577.20	61.50	573.30	477.25	479.20	44.85	45.60	62.00	568.40
690.90	33.50	700.70	630.15	479.25	49.45	49.15	23.00	674.25
839.40	15.00	853.18	804.15	815.92	42.70	43.25	10.00	853.18
260.70	37.50	258.75	294.00	241.10	43.55	41.30	43.00	262.65
337.50	37.50	343.60	272.25	278.40	46.70	45.95	60.00	331.40
479.25	42.50	504.80	268.20	382.40	43.40	43.70	43.00	471.10
572.05	55.00	908.60	536.35	683.20	44.90	48.40	62.50	572.05
475.20	12.50	554.70	408.95	436.40	46.90	46.75	11.50	479.80
135.60	50.00	122.40	130.00	130.55	42.50	41.60	66.00	137.15
183.50	42.50	203.90	178.45	175.90	35.70	36.05	35.00	178.45
718.90	24.00	922.85	623.05	752.55	47.05	48.40	19.00	735.20
1004.40	16.00	1165.50	945.30	1370.50	46.85	49.40	25.00	986.05

KT-25% TENSION_POST2_PPS_RIGHT ARM	KT-25% TENSION_POST2_PPS_ACTUAL PRESSURE_RIGHT ARM	KT-75% TENSION_PrePPT_LEFT ARM	KT-75% TENSION_PrePPT_RIGHT ARM	KT-75% TENSION_PreHPT_LEFT ARM	KT-75% TENSION_PreHPT_RIGHT ARM	KT-75% TENSION_PPS_TARGET PRESSURE_LEFT ARM	KT-75% TENSION_PPS_TARGET PRESSURE_RIGHT ARM	KT-75% TENSION_PrePPS_LEFT ARM
7.50	1041.45	817.85	839.45	46.40	47.55	1019.90	1049.30	7.50
		514.85	481.55	44.55	42.90	643.30	602.10	32.50
17.50	434.45	395.20	434.45	46.65	46.70	494.00	543.00	20.00
2.50	384.45	477.55	556.05	49.95	49.05	597.00	695.00	5.50
62.50	568.40	279.30	305.75	42.65	43.80	349.00	382.00	76.50
25.00	698.75	535.10	520.35	49.70	49.60	669.00	650.00	20.00
20.00	836.02	591.34	450.12	37.80	38.55	739.18	562.65	33.00
39.00	269.50	173.45	209.70	42.25	43.90	217.00	262.00	32.50
40.00	335.50	324.30	285.60	45.55	44.80	405.30	356.90	45.00
47.00	511.90	369.15	356.90	44.60	46.00	461.90	446.60	45.00
60.00	906.55	1465.85	2177.10	49.05	49.65	1831.40	2722.60	55.00
17.50	542.50	450.75	386.45	47.45	46.70	563.40	483.10	22.50
45.00	122.90	108.10	86.70	38.35	38.15	135.10	108.30	62.50
35.00	203.90	142.80	163.20	37.20	40.70	191.20	203.90	35.00
21.50	954.45	589.40	820.90	48.30	49.95	736.70	1025.80	43.00
16.00	1163.50	645.50	792.35	46.85	47.65	806.60	990.10	54.50

KT-75% TENSION_PrePPS_RIGHT ARM	KT-75% TENSION_PrePPS_ACTUAL PRESSURE_LEFT ARM	KT-75% TENSION_PrePPS_ACTUAL PRESSURE_RIGHT ARM	KT-75% TENSION_POST1_PPT_LEFT ARM	KT-75% TENSION_POST1_PPT_RIGHT ARM	KT-75% TENSION_POST1_HPT_LEFT ARM	KT-75% TENSION_POST1_HPT_RIGHT ARM	KT-75% TENSION_POST1_PPS_LEFT ARM	KT-75% TENSION_POST1_PPS_ACTUAL PRESSURE_LEFT ARM
5.00	1019.90	1046.35	1026.75	984.55	45.25	47.05	5.00	1029.70
30.50	639.40	610.00	639.35	575.65	44.30	44.40	24.00	654.10
12.50	516.85	540.35	519.75	531.55	46.40	45.95	25.00	520.75
6.00	601.65	705.10	494.25	550.15	50.20	48.80	5.50	625.20
70.00	395.90	395.45	368.45	358.70	44.15	45.10	65.00	354.80
19.50	685.00	627.20	556.65	588.95	48.60	48.95	29.00	679.15
22.50	745.30	583.49	543.29	461.89	38.20	38.20	25.00	749.23
31.50	235.15	276.35	203.85	230.30	42.60	44.45	38.50	231.00
35.00	407.90	365.06	339.55	298.80	46.50	44.45	60.00	406.85
44.50	460.90	450.70	423.20	459.90	45.50	45.55	41.00	460.95
49.50	1843.15	2681.90	1327.95	1886.45	47.20	48.75	65.00	1799.80
15.00	562.90	493.00	441.55	416.00	48.90	47.35	22.50	558.80
52.50	132.05	105.55	86.70	77.50	37.35	37.00	66.00	131.05
37.50	198.80	203.90	147.90	175.90	35.35	35.70	50.00	198.80
53.50	766.80	1094.15	647.50	818.80	48.05	48.05	33.00	745.45
47.50	805.60	1000.30	870.85	1011.60	42.40	48.05	41.00	807.65

KT-75% TENSION_POST1_PPS_RIGHT ARM	KT-75% TENSION_POST1_PPS_ACTUAL PRESSURE_RIGHT ARM	KT-75% TENSION_POST2_PPT_LEFT ARM	KT-75% TENSION_POST2_PPT_RIGHT ARM	KT-75% TENSION_POST2_HPT_LEFT ARM	KT-75% TENSION_POST2_HPT_RIGHT ARM	KT-75% TENSION_POST2_PPS_LEFT ARM	KT-75% TENSION_POST2_PPS_ACTUAL PRESSURE_LEFT ARM	KT-75% TENSION_POST2_PPS_RIGHT ARM
5.00	1044.40	1076.75	1059.10	45.30	48.45	5.00	1024.80	7.50
20.50	607.00	622.75	579.60	44.65	45.10	21.00	652.15	20.00
12.50	559.95	528.55	466.80	46.85	47.95	17.50	510.90	15.00
5.50	699.25	461.85	546.25	49.35	49.30	8.50	604.10	7.50
60.00	404.75	333.20	369.50	43.85	45.30	62.50	363.55	60.00
26.00	671.30	528.25	560.60	49.00	49.20	20.50	672.25	25.00
17.50	579.60	457.97	461.90	40.50	39.60	22.50	750.21	20.00
40.00	262.65	239.10	203.85	43.65	44.40	42.50	219.55	40.00
30.00	357.90	300.80	333.45	44.35	45.20	42.50	408.95	32.50
40.00	446.65	345.65	447.70	44.80	45.15	45.50	458.85	38.50
55.00	2503.40	1228.75	1792.15	47.35	47.35	67.50	1802.50	60.00
15.00	496.60	439.50	523.15	47.90	46.35	25.00	575.15	12.50
42.50	105.05	94.85	74.45	38.70	37.70	61.50	129.50	47.50
47.50	203.90	178.45	173.35	39.50	37.05	37.50	198.80	35.00
40.50	1061.50	716.85	831.95	47.95	49.70	43.50	744.40	50.50
31.00	999.30	766.85	945.25	45.95	47.80	38.50	808.65	26.00

KT-75% TENSION_POST2_PPS_ACTUAL PRESSURE_RIGHT ARM	NO KT_PrePPT_LEFT ARM	NO KT_PrePPT_RIGHT ARM	NO KT_PreHPT_LEFT ARM	NO KT_PreHPT_RIGHT ARM	NO KT_PPS_TARGET PRESSURE_LEFT ARM	NO KT_PPS_TARGET PRESSURE_RIGHT ARM	NO KT_PrePPS_LEFT ARM	NO KT_PrePPS_RIGHT ARM
1051.30	896.35	1005.15	47.40	49.00	1119.90	1256.20	5.00	5.00
616.85	665.85	622.70	44.85	44.25	831.60	777.70	31.50	41.00
574.70	336.40	380.50	45.50	47.00	420.50	475.60	10.00	12.50
707.55	525.60	498.15	48.05	48.65	657.00	622.70	9.00	6.50
402.80	370.40	336.15	44.25	45.20	461.00	420.00	70.50	73.00
673.25	675.20	608.60	49.10	49.50	844.00	761.00	23.50	26.50
572.70	714.91	632.53	40.80	40.00	999.05	790.66	32.50	31.00
271.45	203.85	299.85	42.25	46.10	255.00	375.00	35.00	40.00
362.00	565.95	685.80	47.25	46.50	706.70	856.60	60.00	47.50
446.65	537.90	629.65	43.55	44.80	672.00	786.20	36.00	33.50
2490.70	532.30	703.60	47.65	49.10	665.90	880.00	57.50	42.00
497.65	397.70	399.70	45.95	47.35	497.10	500.00	10.00	12.00
106.60	113.70	91.80	37.40	38.45	141.70	115.20	67.50	55.00
203.90	160.65	173.40	36.10	39.35	200.80	216.70	35.00	27.00
1083.95	590.40	872.90	47.90	49.20	737.44	1091.10	32.00	43.00
993.20	1141.05	1303.20	48.40	48.10	1425.60	1628.50	38.00	32.00

NO KT_PrePPS_ACTUAL PRESSURE_LEFT ARM	NO KT_PrePPS_ACTUAL PRESSURE_RIGHT ARM	NO KT_POST1_PPT_LEFT ARM	NO KT_POST1_PPT_RIGHT ARM	NO KT_POST1_HPT_LEFT ARM	NO KT_POST1_HPT_RIGHT ARM	NO KT_POST1_PPS_LEFT ARM	NO KT_POST1_PPS_ACTUAL PRESSURE_LEFT ARM	NO KT_POST1_PPS_RIGHT ARM
1125.80	1248.40	997.35	959.10	46.10	47.05	3.00	1118.95	5.00
846.30	830.65	638.45	709.00	45.40	45.65	34.50	833.55	20.50
435.40	477.60	358.90	342.25	45.85	46.15	17.50	430.50	15.00
675.70	647.25	428.55	560.95	48.80	49.05	6.50	687.45	5.00
479.00	430.00	367.50	457.25	44.60	45.70	65.00	464.50	70.00
847.70	743.25	658.60	521.40	49.45	50.40	33.00	847.70	37.00
1028.72	797.28	602.13	634.49	40.25	41.05	32.50	994.39	28.00
263.65	394.95	253.85	238.15	44.50	45.30	31.00	274.40	43.00
739.30	879.55	446.15	624.55	48.25	46.20	50.00	724.00	40.00
667.90	780.10	520.05	713.80	45.05	45.95	31.00	678.10	33.00
673.05	869.80	532.45	664.85	46.85	48.15	61.50	666.90	55.00
518.00	526.20	462.95	333.45	45.85	47.50	12.50	511.90	14.00
139.70	112.70	97.40	90.25	38.50	38.15	61.00	135.65	66.00
203.90	239.60	163.20	188.60	36.30	37.45	42.50	203.90	45.00
755.60	1081.90	670.95	1011.55	47.60	48.80	37.00	754.60	53.00
1393.95	1635.65	1356.20	1372.50	47.05	48.45	34.00	1427.60	37.00

NO KT_POST1_PPS_ACTUAL PRESSURE_RIGHT ARM	NO KT_POST2_PPT_LEFT ARM	NO KT_POST2_PPT_RIGHT ARM	NO KT_POST2_HPT_LEFT ARM	NO KT_POST2_HPT_RIGHT ARM	NO KT_POST2_PPS_LEFT ARM	NO KT_POST2_PPS_ACTUAL PRESSURE_LEFT ARM	NO KT_POST2_PPS_RIGHT ARM	NO KT_POST2_PPS_ACTUAL PRESSURE_RIGHT ARM
1235.60	1073.80	1029.70	47.60	47.40	3.00	1127.80	5.00	1247.40
786.50	550.15	603.15	45.30	46.30	18.50	827.70	27.00	789.45
494.25	366.80	390.30	44.85	49.05	15.00	449.15	10.00	488.40
634.50	503.05	613.85	49.10	48.95	6.00	664.90	5.00	634.50
436.50	345.95	430.20	45.55	45.75	65.00	454.75	63.00	431.20
758.50	555.65	528.20	48.95	48.75	34.00	863.30	34.00	736.95
811.01	672.74	559.96	41.70	40.90	32.50	1017.93	25.00	810.03
404.75	300.85	314.60	45.40	43.90	39.00	262.65	46.00	395.90
879.40	502.25	611.80	46.80	45.40	55.00	721.50	50.00	877.00
772.45	593.95	741.85	45.25	45.10	31.00	673.00	34.00	792.85
874.95	640.35	720.95	46.50	48.80	57.50	668.95	50.00	863.70
512.95	599.60	436.45	47.15	48.00	9.50	519.05	8.00	521.10
113.70	101.45	99.95	38.20	39.65	61.50	138.65	54.00	114.20
239.60	147.90	175.90	37.55	39.15	52.50	203.90	45.00	224.30
1119.65	840.25	970.75	48.40	48.65	23.50	755.65	47.00	1123.75
1635.80	1407.20	1512.20	47.75	48.10	32.00	1406.15	33.00	1630.50

KT= Kinesiotape
Pre=pretest
Post1=posttest 1
Post2=posttest 2
HPT= heat pain threshold
PPT = pressure pain threshold
PPS = pressure pain suprathreshold test
