# Supplementary material for: Effect of different Kinesio tape tensions on experimentally-induced thermal and muscle pain in healthy adults
Source: PLoS One. 2021 Nov 5;16(11):e0259433. doi: 10.1371/journal.pone.0259433 (PMC8570489; doi:10.1371/journal.pone.0259433)
Supplement: S2 File — (PDF) [file pone.0259433.s002.pdf]

Using the scale below please rate your belief in the ability of Kinesio tape to reduce pain. Place a vertical line on the scale to indicate your rating.

**I have no belief in the ability of Kinesio tape to reduce pain**	**I have complete belief in the ability of Kinesio tape to reduce pain.**
